# Hand-Held Ultrasound of the Lung: A Systematic Review

**DOI:** 10.3390/diagnostics11081381

**Published:** 2021-07-31

**Authors:** Mariam Haji-Hassan, Lavinia Manuela Lenghel, Sorana D. Bolboacă

**Affiliations:** 1 Department of Medical Informatics and Biostatistics, Iuliu Hațieganu University of Medicine and Pharmacy Cluj-Napoca, Louis Pasteur Str., No. 6, 400349 Cluj-Napoca, Romania; mariam.haji-hassan@umfcluj.ro; 2 Department of Anatomy, Iuliu Hațieganu University of Medicine and Pharmacy Cluj-Napoca, Louis Pasteur Str., No. 6, 400349 Cluj-Napoca, Romania; 3 Department of Radiology, Iuliu Hațieganu University of Medicine and Pharmacy Cluj-Napoca, Clinicilor Str., No. 3-5, 400006 Cluj-Napoca, Romania; 4 Radiology and Medical Imaging Department, County Emergency Clinical Hospital, Clinicilor Str., No. 3-5, 400006 Cluj-Napoca, Romania

**Keywords:** lung ultrasound, hand-held ultrasound device, lung disease, COVID-19 (Coronavirus Disease 2019)

## Abstract

Background: The ultrasound examination is a surface technique with an accurate diagnosis of pathological processes adjacent to the pleural line. The purpose of the study was to evaluate the role of hand-held ultrasound devices (visual stethoscopes) in the diagnosis of peripheral lung disease. Methods: We conducted a systematic search of literature comparing the diagnostic accuracy of truly hand-held ultrasound devices compared to conventional high-end ultrasound devices, chest X-rays, thoracic CT (computer tomography), or physical examinations to diagnose peripheral lung lesions. ScienceDirect, PubMed, and PubMed Central bibliographic databases were searched within a time limit of 15 years. Results: The applied search strategy retrieved 439 studies after removing duplicates; 34 were selected for full-text review, and 15 articles met all inclusion criteria and were included in the analysis. When comparing hand-held ultrasound devices to chest X-rays, negative predictive values were above 90%, while positive predictive values tended to be lower (from 35% to 75.8%). Hand-held ultrasound reached a correlation of 0.99 as associated with conventional ultrasound with a Bland–Altman bias close to zero. Conclusions: Being accessible, radiation-free, and comparatively easy to decontaminate, hand-held ultrasound devices could represent a reliable tool for evaluating peripheral lung diseases. This method can be successfully employed as an alternative to repeated X-ray examinations for peripheral lung disease monitoring.

## 1. Introduction

Ultrasound is a medical technique that has recently begun to be used for investigating lung disease, which was a surprising turn for medical imaging technology [1,2]. For years, the method was thought to be of little use in this area because the air in the lung scatters and impedes the transmission of sound waves. However, the lung’s surface is a strong reflector of ultrasound waves and thus creates several reverberation artifacts. However, these artifacts contain valuable information and correlate with the current lung pathophysiology [3].

Expert recommendations support the use of ultrasound examination for a vast array of lung diseases such as pleural effusions, interstitial pulmonary lesions, lung consolidations, etc., and in different settings, including at point-of-care [4,5,6,7]. Lung ultrasonography gives results comparable to thoracic computer tomography (CT) scanning while having the advantages of portability, repeatability, low cost, and absence of irradiation [8]. Coronavirus Disease 2019 (COVID-19) generally begins in the terminal alveoli, close to the pleura, and can be clearly observed by lung ultrasound [9]. Lung ultrasound is a surface imaging technique useful in cases of acute respiratory failure, and bedside US should be performed for the early diagnosis of COVID-19 pneumonia in all the patients who present to the emergency department with flu-like symptoms in the era of novel COVID-19 [10].

Several hospitals in COVID hotspots are using hand-held ultrasound. Using these systems, they are examining patients to determine whether they should be admitted and, if so, whether they need intensive care [11]. The absence of ionizing radiations means doctors can use the devices every day to closely track the course of the disease. On the other hand, auscultation presents a high risk of nosocomial transmission because a stethoscope cannot be covered entirely by protective equipment [12].

Several organizations, such as the Canadian Association of Emergency Physicians, and experts recommend using a wireless probe and tablet as the most appropriate ultrasound equipment, as they can be easily wrapped in single-use plastic covers to reduce contamination and promote sterilization. It is possible to put a sheath around the entire system to prevent any pathogens from contaminating it [13,14,15,16].

No systematic review regarding the accuracy of hand-held ultrasound devices for scanning peripheral lungs diseases has previously been conducted. Considering the recommendations to use hand-held ultrasound devices because of their portability and reduced risk of contamination, we performed a systematic search of the literature to assess pocket-size devices’ performance as a diagnostic method for lung pathology.

## 2. Materials and Methods

This review was performed in accordance to the PRISMA (Preferred Reporting Items for Systematic Reviews and Meta-Analyses) guidelines. The study protocol was registered in PROSPERO with the registration number CRD42021242620.

### 2.1. Search Strategy

The search strategy was constructed to address the following:

- Problem: lung disease
- Intervention: hand-held (pocket-sized) ultrasound (US)
- Comparisons: X-ray OR conventional US OR computer tomography (CT)
- Outcome: accuracy
- Study design: any

We conducted the literature search in PubMed, ScienceDirect, and PubMed Central (PMC) bibliographic databases, from 2006 to 8 April 2021, covering the 15-year period that marked hand-held ultrasound devices’ appearance and development. The search strategy was designed and carried out with input from all investigators. 

The following string was used in searching the PubMed database: (pneumonia[Title/Abstract] OR lung[Title/Abstract] OR respiratory OR coronavirus[Title/Abstract] OR COVID-19[Title/Abstract])[Title/Abstract] AND (portable[Title/Abstract] OR hand-held[Title/Abstract] OR handheld[Title/Abstract] OR pocket-size[Title/Abstract] OR visual stethoscope)[Title/Abstract] AND (ultrasound[Title/Abstract] OR ultrasonography[Title/Abstract] OR visual stethoscope)[Title/Abstract].

### 2.2. Study Selection

Our predetermined list of exclusions included:

(1) non-English study;
(2) impossible to obtain the full-text considering the university subscription to bibliographic databases or the request submitted to the first or the corresponding author, or payment for the full-text article;
(3) article type (e.g., opinion of the experts, editorials, case reports, abstracts, commentary, book chapters, etc.);
(4) not truly-portable ultrasound devices or portable US devices (at the size of a laptop);
(5) no comparison of hand-held ultrasound examination with physical examination, biological testing or other imaging methods (e.g., X-ray, conventional US, computer tomography (CT), etc.);
(6) lung ultrasound performed by non-doctors (e.g., medical students, nurses) or using portable US devices (small devices compared to the console-style ultrasound machines that can be carried out by hand);
(7) the studies not applied to human subjects. Duplicated studies were excluded during the screening.

The articles’ selection involved a two-step screening process and was conducted after eliminating the duplicated titles using the Conditional Formatting features of Microsoft Excel. In the first step of screening, two independent researchers screened each article’s titles and abstracts for relevancy to hand-held US screening of peripheral lung diseases. In the second step, the full texts were screened by two independent researchers who assessed the studies for inclusion. In both screening steps, any disagreement was resolved by the intervention of a third independent reviewer, if necessary. 

Articles that described hand-held lung ultrasounds performed by non-doctors (e.g., medical students, nurses), those that referred to veterinary medicine, or those that involved were excluded.

### 2.3. Data Extraction

Data extraction was performed using a self-developed extraction form. The following data were extracted independently by two individual researchers:

(1) study settings (e.g., when?, where?);
(2) study design (e.g., target condition, index test, comparative test, methods to estimate the accuracy of the hand-held US, etc.);
(3) study results (e.g., eligible subjects, evaluated subjects, characteristics of the evaluated patients, accuracy parameters).

### 2.4. Quality of Reporting Assessment

The quality of the studies included in the systematic review was evaluated by the QUADAS-2 tool (Quality Assessment of Diagnostic Accuracy Studies 2) [17].

### 2.5. Presentation of the Findings

The results were reported using a narrative method: tabulation of the study characteristics (e.g., year, country/region, condition studied, etc.), diagnosis methods (index test and comparator test), characteristics of the sample (e.g., number of participants, age, gender), and reported accuracy methods. The evaluated risk of bias and applicability was conducted for the following domains: patients selection, index test, reference standard, flow and timing.

## 3. Results

**Figure 1:**
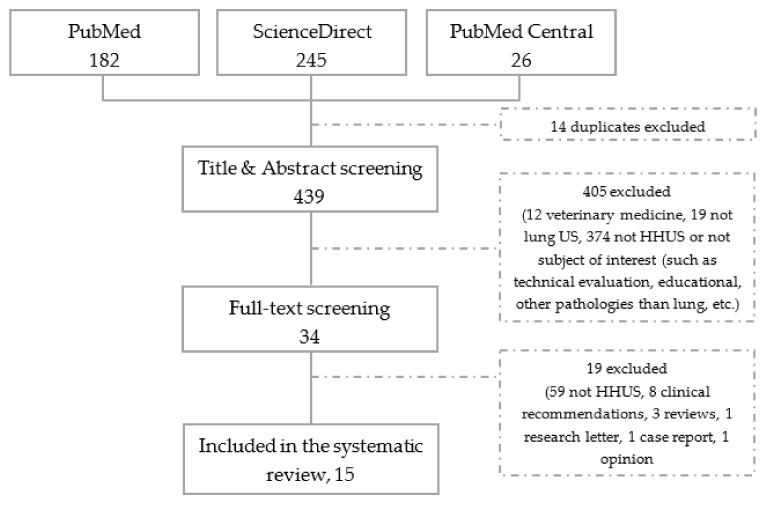
Flowchart of the literature screening (HHUS = Hand-Held UltraSound).

Four hundred and fifty-three articles were retrieved from all search bibliographic databases and 15 studies were evaluated (Figure 1): Bedetti et al. [18], Kajimoto et al. [19], Lisi et al. [20], Cogliati et al. [21], Filopei et al. [22], Platz et al. [23], Sforza et al. [24], Phillips and Manning [25], Bobbia et al. [26], Bensted et al. [27], Lima et al. [28], Newhouse et al. [29], Jalil et al. [30] Dini et al. [31], Bennett et al. [32].

Five out of the 15 studies were conducted in Italy [15,17,18,25,32,33] and in most cases, eligible subjects on a specific period of time [23,24,25,26,28,30,31,32] were evaluated with a hand-held US device (Table 1). In the majority of cases, the role of the index test (namely HHUS, hand-held ultrasound) was diagnostic [19,20,21,22,23,24,26,27,30,32,33] followed by screening [28,29,32] and only one study as triage [24]. One of the included studies was a pilot study (Bobbia et al. [26]).

The number of eligible participants were reported by four studies and were equal with 39 (Cogliati et al. 2014 [21]), 37 (Platz et al., 2015 [23]), 198 (Bensted et al., 2018 [27]) and 54 (Newhouse et al. 2020 [29]). The evaluated sample ranged from 18 (Bennett et al. 2021 [32]) to 165 (Bensted et al., 2018 [27]) (Table 2). The characteristics of the evaluated subjects and the reported metrics for assessing the accuracy are summarized in Table 2.

Seven studies included in the analysis had a low risk of bias, and they had a low risk of applicability concerns (Table 3).

## 4. Discussion

### 4.1. Main Findings

The studies included in this systematic review have significant heterogeneity regarding the evaluated pathology, sample size, performance metrics, and comparators but indicate the utility of hand-held lung ultrasound. Less than half (seven studies) of the evaluated studies showed a low QUADAS-2 risk of bias, and in most of the cases, the studies with low risk of bias evaluated less than 30 patients with limited generalizability of reported results. As the hand-held US devices emerge towards the point-of-care examination, these technical solutions must be evaluated to show their clinical utility, relevance, and diagnosis accuracy. 

#### 4.1.1. Hand-held Ultrasound and Physical Examination

Overall, hand-held ultrasound (HHUS) devices have been shown to have superior accuracy when compared with physical examination. Filopei et al. [22] showed that residents, after undergoing a brief training in lung ultrasound, are significantly better at diagnosing acute pulmonary edema, pneumonia, and pleural effusions than when they use physical examination alone. Melbye [33] and Gustafsson et al. [34] also consider that lung auscultation for rales and crackles, as well as other clinical data, are not reliable and do not have sufficient sensitivity nor specificity for identifying patients with lung disease. Gustafsson et al. [34] also showed the reliability of the HHUS in the identification of the pulmonary congestion signs.

#### 4.1.2. Hand-Held Ultrasound and Thoracic Radiography

Philips and Manning [25] found a good concordance between X-rays and lung HHUS for various lung diseases, such as pneumonia, pleural effusions, and interstitial lung diseases. They also found that lung ultrasound might anticipate the resolution of pulmonary edema faster than chest X-ray [25]. Bensted et al. [27] and Kajimoto et al. [19] reported lower positive predictive values opposite to the negative predictive value, which was very high. A possible explanation is that small subpleural lesions can be seen better with the help of ultrasound while being overlooked by chest X-ray, accounting for a higher rate of false positives if using chest X-ray as a comparator. This finding supported the results reported by Bourcier et al. [35], who used a portable ultrasound device and chest X-ray examinations with patients who had already been diagnosed with acute pneumonia (Se = 95% for ultrasound examination, and 60% for the chest X-ray *p* < 0.05; NPV = 65% for lung ultrasound against 25% for radiography, *p* < 0.05). Filopei et al. [22] showed that peripheral lesions (e.g., Kerley B lines, pleural effusions, subpleural consolidations) could be detected by HHUS with higher accuracy than deeper lesions (e.g., chronic broncho-obstructive pulmonary disease). 

In all articles included in this review, only lesions present in the superficial (subpleural) areas of the lung can be visualized with HHUS, which is consistent with prior research. The sensitivity of lung ultrasound may be higher for peripheral lesions than in the case of X-rays, and this makes ultrasound particularly suitable for detecting COVID-19 lesions, which evolve in a centripetal direction (start in the periphery and spread towards the central part of the lung). This means lung involvement in COVID-19 infection could be detected early, possibly with greater sensitivity than with X-ray examination but also with a risk of false positives that needs to be further investigated. These results are in line with the findings reported in a meta-analysis that compared the accuracy of chest X-ray and LUS for diagnosing acute pulmonary edema, reporting that lung ultrasound can diagnose 15 more cases than chest X-ray without an increase in the number of false positives [36]. Future prospective studies would be useful to determine if lung ultrasound in the initial evaluation of suspected acute pulmonary edema patients improves diagnosis, treatment, and outcomes of these patients, even for conventional or pocket-size US devices. 

#### 4.1.3. Hand-Held Ultrasound and Computed Tomography

Thoracic CT is usually seen as the gold standard when investigating lung pathology, but it is not always the first choice because of the risk of radioactive exposure, especially in high-risk groups (e.g., children, pregnant women), and the examination requires high costs [37]. Similarly, critically ill patients whose conditions can change rapidly demand repeated examinations. HHUS has proven high sensitivity, and low specificity for identifying interstitial lung disease when comparing lung HHUS with high-resolution CT and with high concordance between HHUS and CT was high [21]. In the same study, pocket-size ultrasound devices were found to provide a diagnostic yield similar to higher-level devices [21]. Bobbia et al. [26] compared the accuracy of various types of ultrasound probes while using CT as a gold standard with both experienced and novice physicians and find a good concordance only with trained and expert physicians. The agreement between LUS and CT was poor for residents, revealing the necessity for training standards for novices [26]. Buonsenso et al. [38] reported conventional lung ultrasound (LUS) as more sensitive than chest X-ray for diagnosing COVID-19 lesions with a high correlation between LUS and CT on this category of patients.

#### 4.1.4. Hand-Held Ultrasound and High-End Ultrasound

Tight concordance between HHUS and high-end ultrasound evaluations is reported in the scientific literature, even when comparing experts using a high-tech device with beginners using a hand-held ultrasound device [18]. A possible explanation is the fact that lung ultrasound is easier to perform than other types of ultrasound. Furthermore, the HHUS devices incorporate algorithms that guide the users towards the image. Bennett et al. [32] reported an absolute level of bias equal to −0.0016 (995 confidence interval [−0.054 to 0.021], Bland-Altman method) when the LUS score was compared to a pocket-sized ultrasound score on patients with COVID-19. Furthermore, a high concordance was reported between the two methods (0.990, 95%CI [0.980 to 0.995]) [32]. The Bennett et al. [32] study was cited in a NICE (National Institute for Health and Care Excellence) medtech innovation briefing on nice.org.uk [39]. Burleson et al. [40] reported the use of Butterfly iQ+ device in rural east Africa (no comparator) on evaluation of different systems (e.g., pulmonary, cardiac, abdominal, and musculoskeletal). Five emergency physicians evaluated the performance of the Butterfly iQ+. They considered that it performed well and met their needs for point-of-care ultrasound. The use of the same probe for whole-body scanning, good image quality for most indications, and the low cost were the main advantages of the Butterfly iQ+ device. Disadvantages included lower quality for cardiac imaging and frequent overheating, which means the investigator needs to pause the examination to wait for the cooling of the device [40]. In Saudi Arabia, the same device was used by Rajendram et al. [41] to assess the presence of shunts using saline microbubble enhanced transthoracic echocardiography and lung ultrasound, suggesting the usefulness of this combined approach in the identification of the shunt etiology. An ongoing study evaluates the utility of self-administrated LUS under teleguidance from a medical professional of the same device (Butterfly iQ+) on COVID-19 positive subjects [42].

### 4.2. Lung Ultrasound in Systematic Reviews

Trauer et al. assessed the utility of conventional LUS in COVID-19, showing higher sensitivity than chest X-ray and possibly than CT [43]. The systematic review reported by Hew et al. [44] showed high sensitivity (from 0.91 to 1.00) and moderate specificity (from 0.78 to 1.00) of chest ultrasound examination in the identification of radiological consolidations in patients with acute respiratory failure. However, all evaluated studies were with a high risk of bias and thus questionable regarding the applicability. Hew et al. [44] highlighted the necessity to report the time when the ultrasound examination was performed because the more time elapses before performing the ultrasound examination, the more likely it is that the lesions would progress to a detectable extent without improving patient outcome. This boosts ultrasound sensitivity, overstating its utility as an initial test. Additionally, analyzing lung regions instead of lungs as a whole inflates specificity, giving the misleading appearance of increased precision [44]. Further studies should be conducted using patients as the unit of analysis, since they are the unit of clinical management.

### 4.3. Key Advantages of Hand-Held Ultrasound Devices for Lung Scanning

Pocket-size ultrasound devices can be adequately cleaned and disinfected from one examination to the next one. In contrast with a high-end ultrasound device, a smaller machine can be fully covered by a protective sheath that can be changed after each use [45]. The use of CT scans and multiple chest X-rays requires the transportation of the patient to the radiology department, increasing the risk of contamination, while pocket-size ultrasound provides point-of-care examinations [46].

Another advantage of lung ultrasound is the monitoring of the disease progression, enabling repeated examinations at every change of the clinical presentation or evaluating treatment efficacy. This is even more important in vulnerable populations, such as pregnant women and children, in whom exposure to radiation can thus be avoided. The performance of hand-held ultrasound devices for lung imaging offers a solution for the challenges posed by traditional imaging methods.

### 4.4. Technological Advancement in Lung Ultrasound

The developers of conventional and hand-held ultrasound devices currently explore the inclusion of artificial intelligence (AI) to increase image quality and assisted diagnosis. Roy et al. [47] developed and used a deep learning (DP) algorithm to estimate pixel-level segmentation translated in the overall pathological score [16] of LUS images on COVID-19 positive patients. The higher reported accuracy was 0.96, but external validation using a larger database is needed to validate the proposed DP algorithm. Evans et al. [48] reported the LUS evaluation with robotic ultrasound equipment that allows the sonographer to perform an examination using a robotic arm located at the patient’s bedside, enabling a minimal exposure to disease.

Artificial intelligence algorithms were also used to automatically count LUS B lines and consolidations, using the data to calculate a lung score that indicates the severity of infection [11]. The same team is also working on advanced apps for its hand-held systems called Vscan [11] allowing inexperienced users to perform high-quality examinations. Philips created the Anatomically Intelligent Ultrasound suite technology, recreating the optimal version of the diagnostic view, automatically computing measurements three to six times faster than manual methods [49].

### 4.5. Downsides of Hand-Held Ultrasound Devices

Despite their advantages, the hand-held probes also have some disadvantages that must be listed [50]:

- Battery life is reduced (2–3 h), and recharging takes approximately 6 h
- Inability to perform prolonged scanning because of overheating, with temperatures rising after 15–20 min, which freezes all imaging until the device has cooled down
- Inferior image quality reported by a majority of users
- It is impossible to perform a comprehensive ultrasound examination because of the lack of spectral Doppler, tissue Doppler and other specific measurements offered by fully-featured conventional devices. However, this is not necessarily applied for lung ultrasound, which does not usually require advanced US techniques.

### 4.6. Limitations of the Study

This article provides a comprehensive view of the application of hand-held ultrasound devices in the evaluation of lung lesions, comparing this method with imaging techniques used in clinical practice. 

However, some limitations of our study must be listed. First, the used search string did not include all types of lung pathology, limiting the reported findings to the peripheral lung lesions. Thus, the presented results cannot be generalized to all pulmonary diseases. Second, since the technology is still emerging, the number of studies included in the systematic review is limited. Considering the small number of studies and diversity of comparators, the heterogeneity between studies is high. The different parameters that were assessed by each study prevented meta-analysis and limited comparisons between studies. The various lung lesions identified during ultrasound lung examinations, such as B-lines and lung effusion, have relatively low specificity and can be observed in several lung or cardiac diseases. Third, although the risk of bias regarding the patient selection, index test, comparator test, and timing of the investigation was assessed to be low, the QUADAS-2 tool does not evaluate the blinding of the examiners. Unclear or incomplete blinding to clinical or comparator examination may have influenced outcomes. Forth, not all of the hand-held ultrasound devices listed in our study (e.g., Optigo) are still on the market from when the study was conducted. Another device that was used in one of the presented studies, Uscan, was designed for urological examination and not for pulmonary imaging. Some devices, which have been shown to have very good image quality and for which we have not identified studies conducted on lung pathology, are not present in this review. 

## 5. Conclusions

Being accessible, radiation-free, and—due to its small size—theoretically easy to decontaminate, hand-held ultrasound devices could represent a reliable evaluation method for peripheral lung diseases. However, further research is needed to clarify their accuracy, reliability, and reproducibility. In the case of peripheral lung lesions, hand-held ultrasound can be successfully employed as an alternative to repeated X-ray examinations.

## Figures and Tables

**Table 1:** Characteristics of the reviewed studies.

Study, Year [Ref.]	Location	Sign/Disease	Index Test Device	Comparator Test
Bedetti et al., 2006 [18]	Pisa, Italy	ultrasound lung comets (ULC) (extravascular lung water)	Optigo (Philips, Andover, MA, USA) (“low-tech beginner”)	conventional US
Kajimoto et al., 2012 [19]	Tokyo, Japan	pulmonary disease	Vscan (GE Healthcare, Japan)	chest X-ray
Lisi et al., 2012 [20]	Siena, Italy	pleural effusion	Vscan, Horten, Norway (1.7–3.8 MHz)	chest X-rays
Cogliati et al. 2014 [21]	Milan, Italy	interstitial lung disease in patients with rheumatoid arthritis	VSCAN, GE Healthcare, Fairfield, CT, USA) supporting a phased array transducer (1.7–3.8 MHz).	conventional US, high-resolution CT
Filopei et al., 2014 [22]	New York City, NY, USA	Dyspnea *	Vscan; GE Vingmed Ultrasound, Horten, Norway	chest X-ray, CT
Platz et al., 2015 [23]	unclear	pulmonary edema—heart failure	VScan, General Electric	conventional US
Sforza et al., 2017 [24]	Italy	acute or worsening of chronic dyspnea	Vscan (General Electric Healthcare	chest X-ray
Phillips and Manning, 2017 [25]	Boston, MA, USA	interstitial edema, pleural effusion, pneumonia, and the presence of a central line	Vscan (GE Vingmed Ultrasound A/S, Horten, Norway)	chest X-ray and conventional transthoracic echocardiography (TTE)
Bobbia et al., 2018 [26]	France	acute respiratory failure	Vscan Dual probe, GE Hea	CT, conventional US
Bensted et al., 2018 [27]	Sydney, Australia	pneumothorax after transbronchial lung biopsy	Vscan; GE Healthcare, Chicago, IL, USA)	chest X-ray
Lima et al., 2019 [28]	Nepal	subclinical pulmonary edema on healthy volunteers	iViz (FUJIFILM SonoSite, Inc)	Ancillary measurements included pulse oximetry (SpO2; %), heart rate (HR; /min), and blood pressure (BP; mmHg)
Newhouse et al. 2020 [29]	Adelaide, SA, Australia	unilateral pleural effusion	Signostics Uscan© hand-held US	conventional US
Jalil et al., 2020 [30]	TX, USA	SARS-CoV-2 pneumonia	Vscan Extend Dual Probe, GE	chest X-ray, SARS-CoV-2 PCR testing
Dini et al. 2020 [31]	Pavia, Italy	COVID-19 pneumonia	CERBERO (ATL, Milano, Italy)	nasopharyngeal swab testing for COVID-19
Bennett et al. 2021 [32]	Siena, Italy	COVID-19 pneumonia	Butterfly iQ	conventional US

* (1) exacerbation of chronic obstructive pulmonary disease or asthma (COPD/asthma), (2) acute pulmonary edema (APE), (3) pneumonia (PNA), (4) pulmonary embolus (PE), (5) pneumothorax (PTX), (6) pleural effusion (PLEFF), and (7) other (OTH), namely anemia, ascites, and dehydration.

**Table 2:** Demographics of the evaluated subjects and reported accuracy metrics.

Study, Year [Ref.]	Sample Size	Age, Years	Men	Accuracy Metric
Bedetti et al., 2006 [18]	20	66 ± 12	18 (90.0)	Feasibility: 100%; Mean ULCs: 35.7 ± 25.3 (“high-tech veteran”) vs. 34.2 ± 26.8 (“low-tech beginner”)
Kajimoto et al., 2012 [19]	90	78.1 ± 9.9	45 (50.0)	Se = 96.2%, Sp = 54.0%, NPV = 90.9%, PPV = 75.0%,
Lisi et al., 2012 [20]	73	68.4 ± 9.2	41 (56.2)	AUC = 0.99, Se = 91.7%, Sp = 99.9% for PE (pleural effusion) >100 mL
Cogliati et al., 2014 [21]	29	64.87 ± 9.9 *n* = 39	10 (25.6) *n* = 39	LUS using a PS-USD: Se = 89% (95%CI = [68 to 100]), Sp = 50% [28 to 72], *n* = 29
Filopei et al., 2014 [22]	69	69 ± n.a.	(52.2)	Se = 36% and Sp = 100% for pneumonia—focus training group vs. Se = 89% and Sp = 100% for pulmonary edema—extended training group
Platz et al., 2015 [23]	21	73 (36 to 86)	17 (81)	number of B-lines: Wilcoxon signed-rank test
Sforza et al., 2017 [24]	68	78 ± 12	43 (62)	Se = 92.6% [74.2 to 98.7], Sp = 80.5% [64.6 to 90.6], PPV = 75.8% [57.4 to 88.2], NPV = 94.3% [79.5 to 99], Acc = 85.3% for interstitial syndrome / effusion
Phillips and Manning, 2017 [25]	64 *	70 ± 15	n.a.	Concordance PS-USD—X-rays: 80% (interstitial edema), 77% (pleural effusion), 92% (pneumonia), 81% (central line)
Bobbia et al., 2018 [26]	10	62 ± 16	5 (50)	Kappa coefficient PUD—CT using vascular probe (6–14 MHz): 0.62 [0.37 to 0.86] for experts and 0.68 [0.44 to 0.91] for trained physicians
Bensted et al., 2018 [27]	165	44 (mean value)	80 (48.5)	Se = 75%, Sp = 93%, PPV = 35%, NPV = 99%
Lima et al., 2019 [28]	20	24.7 ± 5.8	9 (45)	B-line Scores at different altitude: 3 (1400m), 0 (3440 m), 1 (3820 m), 17 (4240m), 12 (5160m)
Newhouse et al., 2020 [29]	53	72.9 ± 12.7	27 (50)	No accuracy assessment; image ratings
Jalil et al., 2020 [30]	69	64 ± n.a.	34 (49)	LUS vs. RT-PCR testing: Se = 91%, Sp = 86%, PPV = 86%, NPV = 91%
Dini et al., 2020 [31]	150	88 ± n.a.	n.a. (15)	Se = 79% in predicting positive naso-pharyngeal testing, Sp = 57%, PPV = 74%, NPV = 62%
Bennett et al., 2021 [32]	18	77.6 ± 10	14 (n.a.)	PS-USD vs. conventional US: Pearson correlation coefficient 0.99, Bland–Altman bias close to zero (absolute level of bias—0.016)

Age is reported as mean±standard deviation or median (range), where range = (first quartile to thirs quartile); n.a. = not available; Data regarding the men are reported as number (%); 95% confidence intervals [lower bound to upper bound]; * 108 examinations; ULCs = ultrasound lung comets; Acc = accuracy; Se = sensitivity; Sp = specificity; NPV = negative predictive value; PPV = positive predictive value; PUD = pocket ultrasound device; LUS = lung ultrasound; PS-USD = pocket-size ultrasound device; CT = computed tomography; RT-PCR = reverse transcriptase-polymerase chain reaction.

**Table 3:** QUADAS-2 quality assessments of evaluated studies.

Study, Year [Ref]	Risk of Bias				Applicability Concerns			Comments
	Patient Selection	Index Test	Comparator test	Flow and Timing	Patient Selection	Index Test	Comparator Test	
Bedetti et al., 2006 [18]	unclear	unclear	unclear	low	low	low	low	
Kajimoto et al., 2012 [19]	unclear	low	low	low	low	low	low	
Lisi et al., 2012 [20]	low	low	low	low	low	low	low	X-ray followed by hand-held US
Cogliati et al., 2014 [21]	low	low	low	low	low	low	low	A short-trained physician, who underwent two sessions of 3h each for recognition of B-lines
Filopei et al., 2014 [22]	low	high	low	low	low	low	low	Brief report
Platz et al., 2015 [23]	low	low	low	low	low	low	low	
Sforza et al., 2017 [24]	low	low	low	low	low	low	low	
Phillips and Manning, 2017 [25]	low	low	unclear	unclear	low	low	low	Different results reported in the abstract and in the body of the manuscript
Bobbia et al., 2018 [26]	low	high	unclear	low	low	low	low	
Bensted et al., 2018 [27]	low	low	low	low	low	low	low	17 patients with pneumothorax
Lima et al., 2019 [28]	low	low	low	low	low	low	low	
Newhouse et al., 2020 [29]	low	low	low	low	low	low	low	
Jalil et al., 2020 [30]	high	low	low	low	low	low	low	
Dini et al., 2020 [31]	low	low	low	unclear	low	low	low	
Bennett et al., 2021 [32]	unclear	low	low	low	low	low	low	Two different operators, both experts in lung ultrasound
